# Speciality Grand Challenge for “Biofilms”

**DOI:** 10.3389/fcimb.2021.632429

**Published:** 2021-02-22

**Authors:** Christophe Beloin, Diane McDougald

**Affiliations:** ^1^ Genetics of Biofilms Laboratory, Institut Pasteur, UMR CNRS2001, Paris, France; ^2^ iîhree lnstitute, University of Technology Sydney, Sydney, NSW, Australia; ^3^ Singapore Centre for Environmental Life Sciences Engineering, Nanyang Technological University, Singapore, Singapore

**Keywords:** biofilm infections, multi-species biofilm, infection models, biofilm detection methods, biofilm heterogeneity

## Introduction to Biofilms - Properties of Biofilms

The idea that most bacteria in nature are attached to surfaces was recognized as early as 1933 when Henrici stated after investigating fresh-water bacteria that “it is quite evident that for the most part water bacteria are not free floating organisms, but grow attached upon submerged surfaces” (Henrici, 1933). Zobell followed up with work that demonstrated seawater biofilms precede the attachment of other macro-fouling organisms (Zobell and Allen, 1935). Thus, began this field of research which was furthered by work on biofilms occurring in nature and the growing realization that a number of infections are biofilm-related (Costerton et al., 1999; Hall-Stoodley et al., 2004).

### Biofilm Life Cycle

Biofilms are structurally diverse and dynamic (Hall-Stoodley et al., 2004). Biofilm formation occurs in stages beginning with the attachment of a cell on the surface, followed by division and secretion of extracellular polymeric substances (EPS) and formation of microcolonies (Costerton et al., 1987). There are channels that allow for exchange of nutrients and cellular waste between the microcolonies. The final stage of the biofilm lifecycle is dispersal which is driven by, but not limited to environmental cues and bacterially derived signals (reviewed in (McDougald et al., 2011). These signals include the second messenger cyclic-di-GMP (Jenal et al., 2017), nitric oxide (Barraud et al., 2009) and quorum sensing (Rice et al., 2005).

### Increased Resistance to Antimicrobials and Matrix Properties

Biofilms are more resistant to a variety of stresses than their planktonic counterparts. For example, biofilms have been reported to be up to 1000-fold more resistant to antibiotics than planktonic cells (Costerton et al., 1999) as well as showing increased resistance to other environmental stresses and predation by protists (Matz et al., 2005). This increased resistance is due to changes in cellular metabolism of biofilm cells compared to planktonic cells (Lebeaux et al., 2014; Crabbé et al., 2019). It is also due to the physical protection provided by the extracellular matrix (Flemming and Wingender, 2010).

The biofilm matrix is composed of a variety of biopolymers, including polysaccharides, proteins, nucleic acids, lipids, and other biopolymers such as humic substances (Flemming and Wingender, 2010). These components can vary widely between different bacterial species. The polysaccharides and proteins help to protect biofilm cells from antimicrobials by acting as a barrier to their penetration into the biofilm. *Pseudomonas aeruginosa* requires extracellular DNA or eDNA to form a biofilm and for motility within the biofilm (Whitchurch et al., 2002; Gloag et al., 2013). It is this matrix that lends stability to the biofilm making them difficult to remove. The matrix can also bind extracellular enzymes making them available to biofilm cells and sequester other damaging compounds.

## Challenges in Methods for Investigating Biofilms

### From Monospecies to Polymicrobial Biofilms

Since the early observation of biofilms in their natural environments, notably in rivers, researchers have realized the heterogeneity and complexity of biofilm composition and structure. After a long period during which monospecies biofilms have been studied with success we have moved towards the study of polymicrobial biofilms, which are also relevant in multiple biofilm-associated infections in animals and humans and often at the origin of chronic infections (Peters et al., 2012; Wolcott et al., 2013). Among the multitude of *in vitro* and *in vivo* models of biofilm formation and infection models that have been developed (Coenye and Nelis, 2010; Lebeaux et al., 2013; Brackman and Coenye, 2016) some specific models are dedicated to polymicrobial biofilms (Gabrilska and Rumbaugh, 2015; Tay et al., 2016). The relevance of studying polymicrobial biofilms was demonstrated by the importance of the interactions of micro-organisms for the development of biofilms in the context of infection, notably through the cross-talk between species and their impact on mechanisms of antimicrobial recalcitrance (Kragh et al., 2016; Liu et al., 2016; Orazi and O’Toole, 2019; Ibberson and Whiteley, 2020). Further developments of such models are essential to uncover the molecular mechanisms beyond the specific behavior of micro-organisms during such polymicrobial biofilms.

### Tackling the Heterogeneity of Biofilms

In addition to the complexity of polymicrobial biofilms, one hallmark of biofilms in general is their heterogeneity in terms of local environmental conditions that translates into a heterogeneity of physiology of biofilm cells. While studies using technologies such as transcriptomics, proteomics and metabolomics have been successfully applied to image the global physiology or responses to stress of biofilms cells (Seneviratne et al., 2020), there is a clear need to think singularity and to develop technologies that would allow for the study of the physiology of individual biofilm cells and to understand the social interactions within biofilms (Rode et al., 2020). These studies can be performed with the improvement of methods such as single-cell RNA transcriptomics (Blattman et al., 2020; Imdahl et al., 2020) and technologies used to isolate cells from biofilms (Ma et al., 2019). Such recently developed technologies have been successfully applied to image the physiology of *S. aureus* persister cells within macrophages and should be applied hopefully to biofilms. Imaging technologies have also recently improved to locate and trace individual cells within biofilms. Multiplex FISH technology has been applied to characterize the biogeography of the oral microbiota (Valm et al., 2012; Valm, 2019) while recent microscopy imaging and analyzing technologies allowing for dynamically tracking single biofilm bacteria (Hartmann et al., 2019), for example upon antibiotic stress in *V. cholerae* (Díaz-Pascual et al., 2019). Additionally, some very promising tools have been recently adapted to metabolite identification at the single cell level in biofilm, for instance using Raman spectroscopy, Mass spectrometry or electro-chemical chip/fluorophores (Baig et al., 2016; Bellin et al., 2016; Bodelón et al., 2016; Schiessl et al., 2019; Geier et al., 2020; Yang et al., 2020). The upcoming challenge will be, in addition to further development of these technologies, to adapt them to direct observation or characterization of *in situ* biofilms and to models, whether *in vitro*, *ex vivo*, or *in vivo*, that better feature real *in situ* biofilms.

### Going Toward a Better Understanding of *In Vivo* Biofilms: Improving Detection and Observation of *In Situ* Biofilms and Developing Novel Models?

Today there is absolutely no doubt that biofilms occur *in vivo*, especially in compromised hosts, with almost 80% of human infections now recognized to be biofilm-related (NIH, 2002). Biofilms form in body compartments as well as attached to cells and indwelling devices (Bjarnsholt et al., 2013). One well-studied example of this is the infection of cystic fibrosis (CF) patients’ lungs by *P. aeruginosa*. These biofilms are extremely resistant to antimicrobials for reasons discussed above but also due to their ability to resist host defenses. For example, in both *in vitro* and *in vivo* CF models, *P. aeruginosa* was shown to respond to the presence of host immune cells by upregulating a number of quorum-sensing regulated virulence factors such as rhamnolipids which are known to kill immune cells (Alhede et al., 2009). Other common chronic infections include chronic otitis media and chronic wounds that tend to be caused by multispecies infections (Burmølle et al., 2010). Other major sources of *in vivo* biofilms are indwelling medical devices such as intravenous or urinary catheters, pacemakers, endotracheal tubes and artificial joints (Lebeaux et al., 2014; Stewart and Bjarnsholt, 2020). Microorganisms commonly associated with medical devices are *Staphylococcus epidermidis, Staphylococcus aureus*, and *P. aeruginosa* (Hall-Stoodley et al., 2004). These biofilms often result in replacement of the device, which for artificial joints is a significant task. The dispersal of these biofilms has been discussed as a potential treatment, however a large number of planktonic cells entering the blood stream may be more dangerous than other treatments. Numerous infections are also related to biofilms in animals, for example in bovine mastitis, pneumonia, liver abscess, enteritis, urinary tract infection, otitis, wound infection, etc. (Abdullahi et al., 2016) and in plants where bacterial and fungal pathogens colonization can be at the origin of different diseases (Bogino et al., 2013; Castiblanco and Sundin, 2016; Motaung et al., 2020).

For decades our vision of how biofilms look has been governed by our knowledge developed from the use of *in vitro* biofilms models. As stated above, the increasing number of biofilms isolated from real infection situations have brought into question this conventional thinking with the recognition that, with some exception, *in vivo* biofilms rather correspond to patches or cell aggregates that rarely exceed 200 µm (Bjarnsholt et al., 2013) with important consequences on how biofilms finally interact with the host (Alhede et al., 2020). These observations have led to several important statements: in most cases, *in vitro*, and sometimes *in vivo* biofilms models do not relevantly recapitulate biofilms in their real infectious context; the diagnosis of the presence of biofilms linked to infection cannot be made *a posteriori* if we want clinicians to adapt their treatment to the presence of a biofilm. With these statements come potential actions: on one side the necessity to improve detection methods that could, for some of them, allow early detection of biofilms in the host and, for others, give a better characterization of the environment of infectious biofilms. Thus, the need to implement some of the *in situ* information into the current *in vitro* and *in vivo* models, and to develop novel models is paramount. It is clear that to this day some effort has been made in these directions, but we will have to do much more to reach these objectives.

We are still trying to identify biofilm biomarkers that could be used not only for detection but also for vaccine development. Important work has been done in the laboratory of the late Prof. Mark Shirliff in this direction notably with a recent demonstration that *S. aureus* orthopedic implant infections could be detected in human sinuvial fluids using an antibody against the manganese transporter MntC (Harro et al., 2019; Harro et al., 2020). Peptide-fluorescently labeled probes have been recently identified and used to specifically label *P. aeruginosa* biofilms *in vivo* (Locke et al., 2020). Additionally, numerous other detection methods are under development and efforts should be made to allow these technologies to translate into the clinic (Achinas et al., 2020; Parlak and Richter-Dahlfors, 2020). Among them, recently there has been the successful use of imaging technologies using bioluminescence (Chauhan et al., 2016; Hoffmann et al., 2019; Maiden et al., 2019; Gordon et al., 2020; Kreth et al., 2020; Redman et al., 2020; Van Dyck et al., 2020) or intravital imaging (Thanabalasuriar et al., 2019; Abdul Hamid et al., 2020; Gries et al., 2020; Tian et al., 2020) to study the effect of antimicrobial agents on biofilms or the behavior of immune cells in contact with biofilms in various *in vivo* models of biofilm-related infections.

The concept of the micro-environment of biofilm cells during infection has been well conceptualized in a recent perspective article on chronic wounds where several defined zones can be identified and on which information should be gathered in order to access to a global view of the biofilm infectious micro-environment (Kirketerp-Møller et al., 2020). Ideally, non-destructive methods to sample and analyze this micro-environment using micro-dialysis, micro-probes or electrochemical probes should be adapted to *in vivo* measurement as recently explained in (Røder et al., 2020), however, some of these methods also have applications for biofilm detection.

Improving the models for a better understanding of biofilm formation and biofilm cells behavior is also a major objective that should be reached by researchers in the field. Initiatives to evaluate the available models are welcome and should provide a better view of the current more relevant models with some clues on how to improve them (Cornforth et al., 2020). With the increased pressure to restrict the use of animals in research there is also a dire need to identify surrogate models. While there is some utility to pursue the development of non-mammalian models of biofilm-infection such as *Drosophila melanogaster*, *Galleria mellonella* or *Danio rerio* (Zebrafish) (Lebeaux et al., 2013), identification of *ex vivo* models that recreate the infectious environment should be encouraged. Among recently developed *ex vivo* models are, for example, an *ex vivo* pig lung biofilm model used for understanding antibiotic tolerance of *P. aeruginosa* biofilms (Hassan et al., 2020) or an *ex vivo* murine skin biopsy model to characterize *B. burgdoferi* biofilms, the etiological agent of Lyme disease (Torres et al., 2020). Last but not least with the development of reconstituted organs such as organoids or organ-on-a-chip, one could anticipate that adaptations of these models to the understanding of the physiopathology of biofilm infections, interactions of biofilms with the immune response or biofilm behaviors in the presence of antimicrobials would provide important information relevant to biofilms in infectious contexts (Jimi et al., 2017; Choi et al., 2020; Yuan et al., 2020).

### Major Challenges

As described above, the field of biofilms and interactions with higher organisms has clear challenges for future research. 1) The development of new and improved multi-species biofilm models will allow us to investigate interactions that occur in these biofilms, as most *in vivo* biofilms are multispecies biofilms. This is clearly the next step in understanding biofilms in nature and in disease contexts, and this should come with the use of technologies that would allow probing the infectious micro-environment of biofilms. 2) The development of more relevant models, whether *in vitro*, *ex vivo*, or *in vivo*, to elucidate interactions of biofilms with the host in more detail. These models will be necessary not only for understanding biofilm host interactions but also for the investigation of eradication methods or compounds in a more real setting. 3) The development of imaging and detection technologies that could be used to better visualize and detect biofilms *in situ* and should allow when necessary to access the physiology on individual cells within biofilms. As new methods are developed, using these for biofilm research will generate new knowledge and lead to new areas of biofilm research.

## Author Contributions

CB and DM wrote the manuscript. All authors contributed to the article and approved the submitted version.

## Conflict of Interest

The authors declare that the research was conducted in the absence of any commercial or financial relationships that could be construed as a potential conflict of interest.
